# Indo-Pacific Warm Pool Area Expansion, Modoki Activity, and Tropical Cold-Point Tropopause Temperature Variations

**DOI:** 10.1038/srep04552

**Published:** 2014-04-01

**Authors:** Fei Xie, Jianping Li, Wenshou Tian, Yanjie Li, Juan Feng

**Affiliations:** 1 State Key Laboratory of Numerical Modeling for Atmospheric Sciences and Geophysical Fluid Dynamics, Institute of Atmospheric Physics, Chinese Academy of Sciences, Beijing, China; 2 Key Laboratory for Semi-Arid Climate Change of the Ministry of Education, College of Atmospheric Sciences, Lanzhou University, China

## Abstract

The tropical cold-point tropopause temperature (CPTT), a potentially important indicator of global climate change, is of particular importance for understanding changes in stratospheric water vapor levels. Since the 1980s, the tropical CPTT has shown not only interannual variations, but also a decreasing trend. However, the factors controlling the variations in the tropical CPTT since the 1980s remain elusive. The present study reveals that the continuous expansion of the area of the Indo-Pacific warm pool (IPWP) since the 1980s represents an increase in the total heat energy of the IPWP available to heat the tropospheric air, which is likely to expand as a result. This process lifts the tropical cold-point tropopause height (CPTH) and leads to the observed long-term cooling trend of the tropical CPTT. In addition, our analysis shows that Modoki activity is an important factor in modulating the interannual variations of the tropical CPTT through significant effects on overshooting convection.

The strongest upwelling from the troposphere to the stratosphere occurs over the tropics, and thus the tropics act as the primary channel for tropospheric air entering the stratosphere. However, tropospheric water vapor is freeze-dried near the tropical cold-point tropopause as air crosses the tropopause, leading to very low water-vapor mixing ratios throughout the stratosphere^1^. Both observations and reanalysis data show that interannual variations in the water vapor content of the lower stratosphere is closely associated with anomalies in the tropical CPTT^2^,^3^,^4^,^5^,^6^. In recent years, the structure and variation of the tropical tropopause, and the processes that affect it, have become topics of active research^7^,^8^,^9^,^10^,^11^,^12^. The canonical El Niño–Southern Oscillation (ENSO) and quasi-biennial oscillation (QBO) were the primary sources of interannual variations in the tropical tropopause from 1950 to 1980^2^,^13^,^14^,^15^. However, since the 1980s, the tropical CPTT has shown not only interannual variations but also a decreasing trend^2^. This decrease is thought to be associated with convection anomalies caused by SST variations over the western Pacific, which have previously been shown to be closely related to changes in the CPTT^3^. However, the canonical ENSO and QBO cannot explain the interannual variations in the tropical CPTT since the 1980s^2^.

The maps in Fig. 1 show the correlation between the tropical-averaged CPTT (here, the tropics are defined globally as the zone between 15°N and 15°S) and SST during 1981–2010, based on three datasets: Met Office Hadley Centre SST (HadISST), Kaplan Extended SST (Kaplan SST), and NOAA Extended Reconstructed SST (ERSST). Of the three, only the ERSST data (Fig. 1a) is strongly correlated with tropical CPTT over the western Pacific, with the exception of SST from HadISST and Kaplan SST (Figs. 1b and c). Furthermore, there is no significant correlation between the tropical-averaged CPTT and convection in the region (Fig. 1d). These results suggest that whether the changes in SST over the western Pacific leading to the tropical-averaged CPTT variations remain uncertain. Since the 1980s, the second leading ode of tropical SST variations show a warm center in the central Pacific, with two cold centers, one in the eastern Pacific and one in the western Pacific; this is distinct from the pattern observed during canonical El Niño episodes, which are characterized by a cold center in the eastern Pacific and a warm center in the western Pacific^16^. This distinct pattern is referred to as El Niño Modoki (the negative phase of El Niño Modoki, known as La Niña Modoki, shows a cold center in the central Pacific with warm centers in the eastern and western Pacific). El Niño Modoki events have been increasing in frequency since the 1980s^16^,^17^. Recent studies have found that El Niño Modoki has significant impacts on interannual variations in the troposphere^18^,^19^,^20^. However, it remains uncertain whether interannual variations in the tropical CPTT since the 1980s are related to El Niño Modoki events. These uncertainties, along with observed climate change, motivated the present analysis of the trend and interannual variations in the tropical CPTT since the 1980s.

## Results

An increase (decrease) in the tropopause height corresponds to a cooling (warming) of the tropopause^9^,^21^. The tropical-averaged CPTH shows a strong increasing trend and significant anti-correlation (r = −0.79) with the decreasing tropical-averaged CPTT since the 1980s (Fig. 2a). This suggests that the variations in the tropical-averaged CPTT since the 1980s have largely been determined by the tropical-averaged CPTH. It is interesting that the regions with the largest decrease in tropical CPTT since the 1980s are concentrated over the Indo-Pacific warm pool (IPWP) (Figs. 2b and c). The IPWP area has been increased significantly since 1980^22^,^23^. It implies that the tropical-averaged CPTH trend, which modulates the tropical CPTT trend, is probably associated with IPWP area changes.

To evaluate the potential impact of changes in the IPWP area, we first define a simple energy index (EI), which approximates the enthalpy changes over a region of sea surface, as follows: 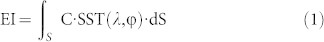
where *S* is the area; *C* is the heat capacity of seawater (4096 J/(kg·K)); *λ* and *φ* are the longitude and latitude coordinates, respectively; and dS = *a*^2^ **·** cosφ **·** dλdφ, where *a* is the radius of the earth. Here, we calculated the EI over the IPWP area, known as EI_(IPWP)_. The IPWP is defined as the area where the SST is higher than 28°C between 30°S–30°N and 50°E–180°E–140°W, based on HadISST data.

The tropical-averaged CPTH and EI_(IPWP)_ are strongly correlated, and both show strong increasing trends since the 1980s (Fig. 3a). However, the linear trends in tropical-averaged CPTH and EI_(IPWP)_ are less distinct between 1950 and 1980, and the correlation between them is weak (Fig. 3b). The 30-year sliding correlations of tropical-averaged CPTH and EI_(IPWP)_ between 1950 and 2010 clearly show that the correlation has steadily strengthened since the 1980s (Fig. 3c). This implies a drift in tropopause changes since the 1980s, possibly related to the areal expansion of the IPWP. Furthermore, IPWP expansion, which leads to variations in the tropical-averaged CPTH, may be an important factor influencing the variations and trends in the tropical-averaged CPTT since the 1980s.

The thermal convection and air expansion caused by a heating source can contribute significantly to an increase in tropopause height^24^,^25^. The IPWP is a heating source, but the question is whether its areal enlargement enhances thermal convection or air expansion to a degree sufficient to increase the tropical CPTH. This question is addressed here through statistical analysis and model simulations. Figure 4a shows time series of tropical-averaged CPTH and the tropical-averaged vertical velocity at 100 hPa (ω_100_), which represents the changes in convection at the tropopause. The CPTH has an evident increasing trend but the ω_100_ does not, and the correlation between them is weak, suggesting that thermal convection may not be the primary mechanism responsible for CPTH lifting. To further investigate the relationship between tropical CPTH lifting and the areal expansion of the IPWP, we perform two time-slice experiments integrated by the CAM3 model. Experiment 1 (E1) is the control experiment and Experiment 2 (E2) is the sensitivity experiment. The SST used in both experiments is the monthly mean climatology for 1986–2010, except that in E2, the IPWP area is expanded by ~25% in the region 30°S–30°N and 50°E–180°E–140°W to simulate the ~25% expansion of the IPWP area that occurred between 1980 and 2010. Figure 4b shows the expanded IPWP area in E2 compared with E1 (for further details regarding the simulations, see the Methods section). Table 1 lists the differences in annual tropical averaged CPTH, CPTT, and ω_100_ between E2 and E1 (i.e., E2 – E1). The results indicate that warm pool area expansion can indeed significantly lift the tropical-averaged CPTH, resulting in the decrease in CPTT. However, the difference in ω_100_ between the simulations is not significant, supporting the notion that enhanced thermal convection may not be the primary cause of tropical CPTH lifting; instead, air expansion caused by enlargement of the IPWP area is likely to be the key factor in lifting the tropical CPTH.

**Figure 1:**
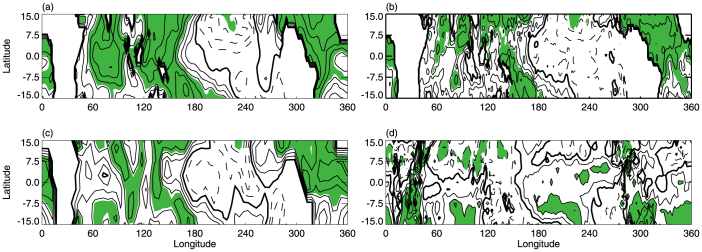
Correlation maps between the tropical averaged CPTT and tropical SSTs. (a) Correlation between tropical-averaged CPTT (15°S–15°N) and ERSST for 1981–2010. Contour interval is 0.1. Solid (dashed) lines represent positive (negative) values; and heavy lines are zero. Correlations significant at the 99% confidence level are shaded (see the Methods section for details regarding statistical tests). (b) and (c) Same as (a), but for HadISST and Kaplan SST data, respectively. (d) Same as (a), but for outgoing longwave radiation (OLR).

Analysis of the correlation between SST and CPTT in each grid cell across the entire tropics for the time series between 1981 and 2010 showed no significant correlation over the western Pacific (Fig. 1). This is because the method considers only the effect of SST on the tropical-averaged CPTT in a single grid cell. However, we considered the total effect of SST in all grid cells throughout the IPWP on the tropical-averaged CPTT. This is why EI_(IPWP)_ has a significant impact on tropical CPTT variations.

**Figure 2:**
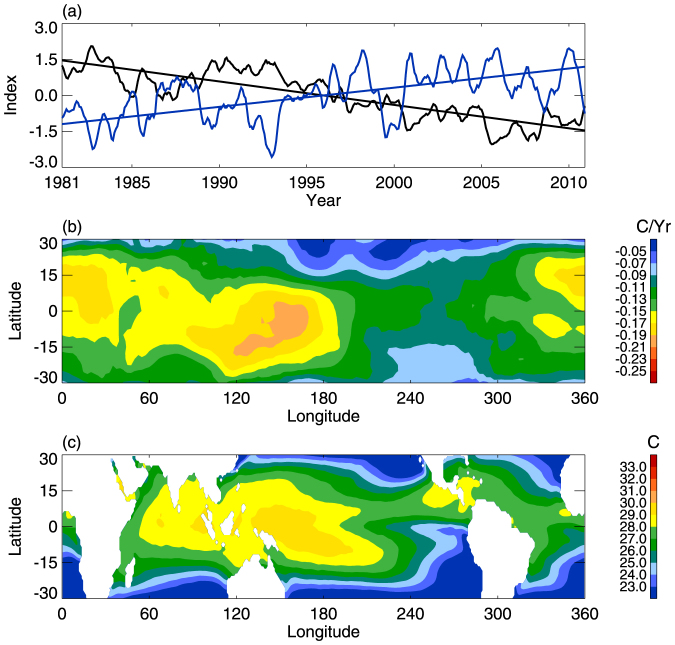
Trend of tropical CPTT. (a) Variations in the tropical-averaged CPTT (black line) and tropical CPTH (blue line). The correlation coefficient between them is −0.79, which is significant at the 99% confidence level. The straight lines are the corresponding linear trends. (b) Spatial distribution of the tropical CPTT linear trend (°C/yr) from 1981 to 2010. (c) Climatological SST distribution (°C) for the period 1981–2010. The white region in (c) is an area of missing values.

An empirical orthogonal function (EOF) analysis of the variability in tropical CPTT shows that the leading principal component (PC1), accounting for 70% of the variance, is associated with tropical CPTH variations, which are themselves related to warm pool area variations (Fig. 5a). Figure 5b shows the EOF1 pattern, which is in agreement with the CPTT trend distribution during the past 30 years (Fig. 2b). PC2, which accounts for 8% of the variance, is significantly correlated with the NINO3 index (Fig. 5c). The EOF2 pattern is consistent with tropical CPTT anomalies during canonical ENSO events (Fig. 5d). PC3 accounts for 6% of the variance, and is strongly correlated with the QBO index (Fig. 5e). The corresponding EOF3 pattern is shown in Fig. 5f. Several previous studies performed EOF analyses on the tropopause temperature. Based on tropical tropopause temperature from ECMWF reanalyses, the leading modes are associated with the canonical ENSO and QBO^26^. However, as we discuss in the Data subsection of the Methods section, the variations in the ECMWF tropopause temperature since the 1980s are not in good agreement with the observations, suggesting that the tropical tropopause temperature in ECMWF reanalyses have biases. A previous study based on NCEP1 data found that the first leading mode of tropical CPTT variations from 1979 to 1999 is associated with canonical ENSO^13^. However, the zonal mean signal was removed from CPTT in that study.

Figure 6a shows that the interannual variations in the tropical-averaged CPTT are anti-correlated with the interannual variations in the tropical-averaged CPTH. However, the correlation coefficient decreases to −0.53 (the correlation coefficient between CPTT and CPTH variations is −0.79, Fig. 2a). This implies that there are factors other than the tropical-averaged CPTH interannual variations that are also important in controlling the interannual variations in the tropical-averaged CPTT. To uncover these processes, we separated the interannual variations of the tropical-averaged CPTT into two parts: one caused by tropical-averaged CPTH interannual variations [CPTT_(H)_], and the other unrelated to tropical-averaged CPTH interannual variations [CPTT_(O)_] (see the Methods section for details). Divergence of the vertical eddy heat flux near the tropopause caused by convection can warm or cool the tropopause, which controls the interannual variations in tropopause temperature^27^. ω_100_ interannual variations are strongly correlated with the interannual variations in tropical-averaged CPTT_(O)_ (Fig. 6b), suggesting that the interannual variations in the tropical-averaged CPTT are caused mainly by an integrated effect of both tropical-averaged CPTH and convection interannual variations (Fig. 6c).

The interannual variations in EI_(IPWP)_ are strongly and significantly correlated with the interannual variations in the tropical-averaged CPTH (Fig. 6d). This implies that the interannual variations in EI_(IPWP)_ modulates the interannual variations in the tropical-averaged CPTH, which then partly affects the interannual variations in the tropical-averaged CPTT (Fig. 6a). The second leading mode of SST tropical variations from 1980 to 2010 relate to the new type of ENSO; namely, El Niño Modoki^16^,^28^. We find that ω_100_ interannual variations are significantly correlated with the Modoki index from 1980 to 2010 (Fig. 6e). El Niño (La Niña) Modoki events correspond to strong (weak) convection, positive (negative) divergence of the vertical eddy heat flux, and a warm (cool) tropopause. That is, El Niño Modoki events result in a positive temperature anomaly in the tropical upper troposphere and tropopause (Fig. 6f), and modulate convection at the tropopause level, which affects interannual variations in the tropical-averaged CPTT. This analysis illustrates that the interannual variations in the IPWP area and Modoki activity may be the primary factors in modulating the interannual variations in the tropical-averaged CPTT.

An EOF analysis of the interannual variability in the tropical CPTT shows that PC1 accounts for 50% of the variance and is associated with the integrated effect of the interannual variations in IPWP area and Modoki activity (Fig. 7a). The EOF1 pattern (Fig. 7b) shows that the maximum anomalies are located in the IPWP region. PC2, which accounts for 10% of the variance, is significantly correlated with the NINO3 index (Fig. 7c). The EOF2 pattern is consistent with CPTT anomalies during canonical ENSO events^2^,^13^,^15^ (Fig. 7d). PC3 accounts for 6% of the variance, and is strongly correlated with the QBO index (Fig. 7e). The corresponding EOF3 pattern is shown in Fig. 7f. The explained variances imply that the integrated effect of IPWP area and Modoki activity on tropical CPTT interannual variations may be more significant than that of the canonical ENSO and the QBO since the 1980s.

Although both canonical El Niño and El Niño Modoki are related to SST anomalies over the tropical Pacific, the present results suggest that El Niño Modoki has a more significant influence on the CPTT than canonical El Niño. Figure 8a shows the monthly SST anomalies, based on HadISST data, over both the equatorial eastern Pacific (5°N–5°S, 150°–90°W), where they are used to define canonical El Niño events, and the equatorial central Pacific (5°N–5°S, 160°E–150°W), where they are used to define El Niño Modoki events. It is apparent that during the past 30 years the amplitudes of SST anomalies are similar overall during the two types of El Niño events, except for 1997. However, the sea surface background temperatures over the two regions are significantly different; specifically, over the equatorial central Pacific they are 28–30°C, but over the equatorial eastern Pacific they are less than 26°C (Fig. 8b). The SST anomalies can cause convective activity to increase sharply over regions with sea surface background temperatures above 26°C^29^,^30^,^31^,^32^, which implies that convective activity is much stronger during El Niño Modoki events than during canonical El Niño events. Figures 8c and 8d show the composite anomalies of the overshooting number, which can reach 15 km (close to the tropopause level in the tropics) during both El Niño Modoki and canonical El Niño, according to Tropical Rainfall Measuring Mission (TRMM) observational data (the calculation of the overshooting number and composite anomalies is explained in the Methods section). The composite results are in agreement with the above analysis. The different intensities of convection give rise to different divergences of the vertical eddy heat flux, which lead to differences in adiabatic heating at the tropopause. This may be the reason that El Niño Modoki has a more significant influence on the tropical CPTT than canonical El Niño. It is important to note that the patterns of deep convection anomalies are similar during the two types of El Niño event^20^. This suggests that both El Niño types generate similar levels of lower-middle tropospheric convection, while El Niño Modoki generates more overshooting convection.

## Discussion

From the present analysis, we indicate that the variations in the tropical-averaged CPTT since the 1980s have been caused mainly by an integrated effect of the areal expansion of the IPWP and El Niño Modoki activity. The impact of this integrated effect on the tropical-averaged CPTT may be more significant than that of the canonical ENSO and the QBO from 1980 to 2010. The continuous areal expansion of the IPWP during the past three decades has caused the decreasing trend of the tropical-averaged CPTT. This expansion represents an increase in the total heat energy of the IPWP that is available to heat the troposphere, likely causing expansion of the air, lifting of the tropical CPTH, and a cooling trend in the tropical CPTT. The data analyzed here suggest that the interannual variations in the tropical-averaged CPTT are caused mainly by interannual variations in the IPWP area and Modoki activity. Modoki events can evidently change the distribution and intensity of overshooting convection, which influences the divergence of the vertical eddy heat flux^27^. This in turn changes the temperature in the upper troposphere and tropopause.

**Figure 3:**
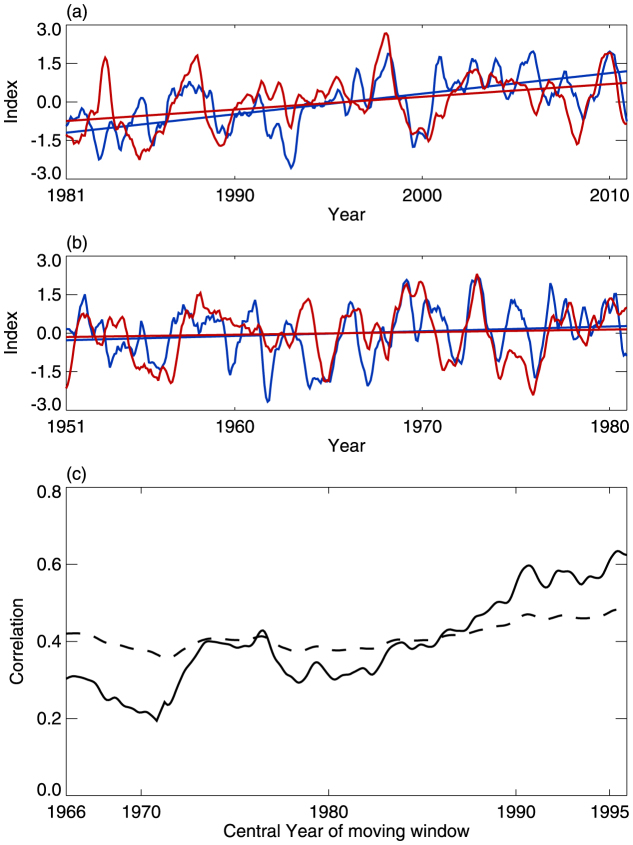
Tropical averaged CPTH vs. EI_(IPWP)_. (a)Variations in the tropical averaged CPTH (blue line) and EI_(IPWP)_ (red line) from 1981 to 2010. The straight lines are the corresponding linear trends. The correlation coefficient is 0.65, which is significant at the 99% confidence level. (b) Same as (a), but for 1951–1980. The correlation coefficient is 0.37, which is not significant at the 95% confidence level. (c) The 30-year sliding correlations of tropical averaged CPTH and EI_(IPWP)_ during 1950–2010. The dashed line shows the 99% confidence level.

**Figure 4:**
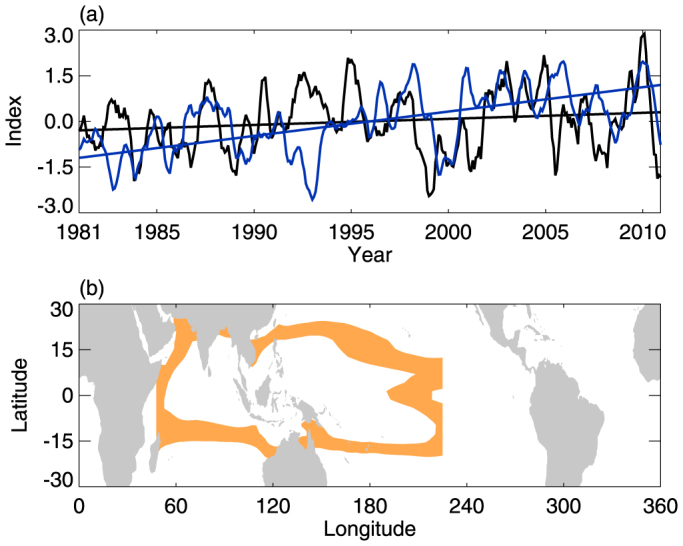
Tropical ω_100_ vs. CPTH and SST forcing. (a) Variations in ω_100_ (black line) and tropical-averaged CPTH (blue line) from 1981 to 2010. The straight lines are the corresponding linear trends. The correlation coefficient is −0.19, which is not significant at the 95% confidence level. (b) The orange area is the expanded IPWP area in E2 compared with E1. The gray region is an area of missing values.

**Figure 5:**
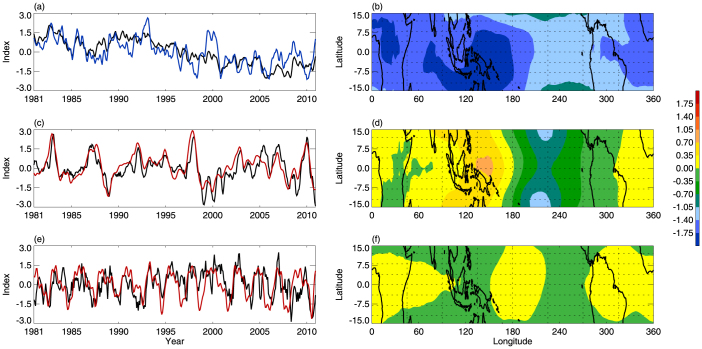
PCs and EOFs of CPTT variability. (a) Variations in PC1 (black line) and tropical-averaged CPTH (blue line). (b) EOF1 pattern. (c) PC2 (black line) and NINO3 index (red line). (d) EOF2 pattern. (e) PC3 (black line) and QBO index (red line). (f) EOF3 pattern. All PCs and EOFs were calculated from EOF analysis of variability of tropical CPTT variations for 1981–2010. The square root of the cosine of latitude is used as a weighting function in EOF analysis.

**Figure 6:**
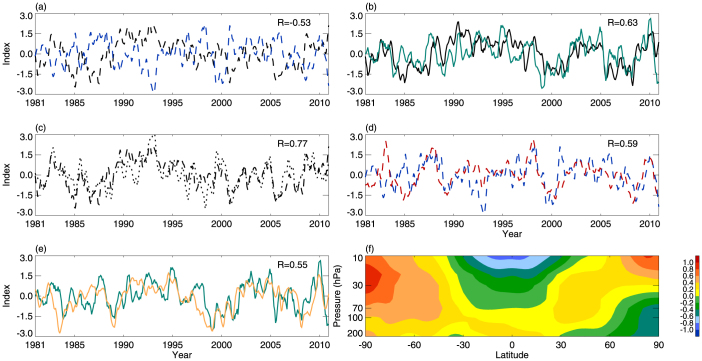
Interannual variations in the tropical-averaged CPTT. (a) Interannual variations in the tropical-averaged CPTT (black dashed line) and CPTH (blue dashed line). (b) The CPTT_(O)_ (black dotted/dashed line) and ω_100_ interannual variations (green line). The CPTT_(O)_ is the CPTT interannual variations with the CPTH interannual variations signal removed. (c) Interannual variations in the tropical-averaged CPTT (black dashed line) and the CPTT_(R)_ (black dotted line). The CPTT_(R)_ was calculated by fitting the interannual variations in the tropical-averaged CPTH and ω_100_ to interannual variations in the tropical-averaged CPTT. CPTT_(R)_ represents the integrated effect of interannual variations in the tropical-averaged CPTH and ω_100_. (d) Interannual variations in the tropical-averaged CPTH (blue dashed line) and EI_(IPWP)_ (red dashed line). (e) The ω_100_ interannual variations (green line) and Modoki index (orange line). The corresponding correlation coefficients are shown in the top-right corners of each panel and all (a–e) are significant at the 99% confidence level. (f) Meridional cross-sections of zonal mean temperature interannual variations, linearly regressed onto the Modoki index for 1981 to 2010. Temperature regressions are shown as local percentage variations, with contour intervals of 0.2%/Modoki index.

**Figure 7:**
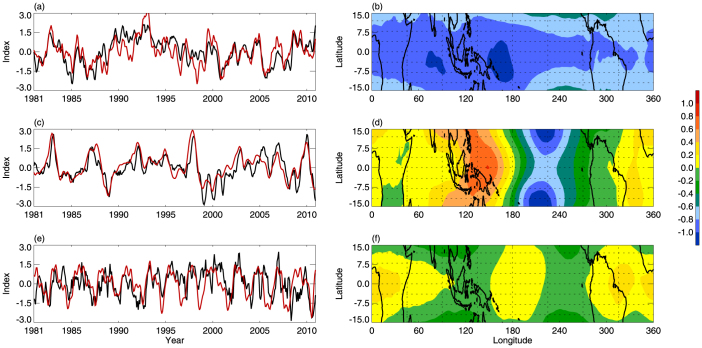
PCs and EOFs of CPTT interannual variability. (a) Interannual variations in PC1 (black line) and CPTT_(R)_ (red line). (b) EOF1 pattern. (c) PC2 (black line) and NINO3 index (red line). (d) EOF2 pattern. (e) PC3 (black line) and QBO index (red line). (f) EOF3 pattern. All PCs and EOFs were calculated from EOF analysis of variability in tropical CPTT interannual variations for 1981–2010.

**Figure 8:**
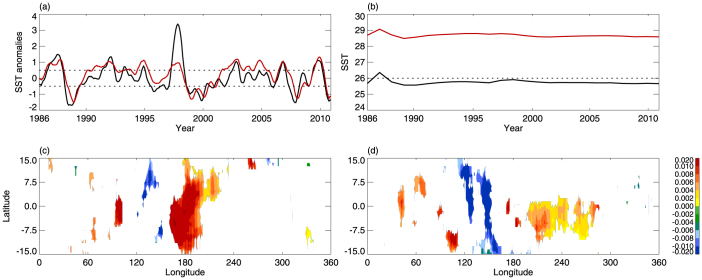
SST changes over the equatorial Pacific and Pacific, and overshooting number anomalies. (a) Monthly SST anomaly changes of the equatorial eastern Pacific (5°N–5°S, 150°–90°W) and equatorial central Pacific (5°N–5°S, 160°E–150°W) based on HadISST. Dashed lines represent the ±0.5°C lines. (b) Same as (a), but for yearly changes in SST. Dashed lines represent the 26°C line. (c) Composite overshooting number anomalies, which can reach 15 km, from TRMM observational data for El Niño Modoki events. Only the anomalies that are significant at the 99% confidence level (Student's *t*-test) are shown. (d) Same as (c), but for canonical El Niño events.

Our findings suggest that tropical CPTT changes are closely related to climate change. Within the context of present and future global warming, the IPWP area is expanding^22^,^23^ and the frequency of Modoki events is increasing^16^,^28^. The implication, therefore, is that IPWP expansion and the occurrence of Modoki events will continue to be the main factors affecting the trend and interannual variations of the tropical CPTT. This study will be helpful for the prediction of future changes in the tropical CPTT, and will provide a good understanding of the variations in future stratospheric water vapor. This study focused on the effect of changes in the areal extent of the IPWP on CPTT. However, further work, looking in detail at the controls on variations in the IPWP and considering why the area of the IPWP changes (see Fig. 3a) is still needed.

## Methods

In this study, the variations are calculated by removing the seasonal cycle from the original time series. Interannual variations are calculated by removing the linear trend and seasonal cycle from the original time series. All time series have been normalized and three-point running averages have been obtained.

### Data

**Figure 9:**
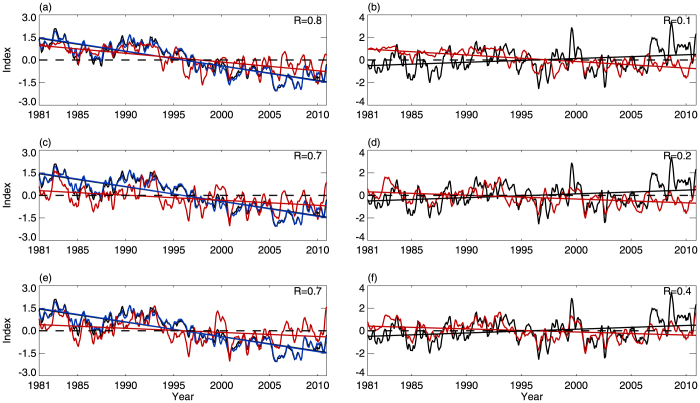
Tropical-averaged CPTTs. Tropical-averaged CPTTs from reanalyses versus those from observations, for 1981 to 2010. (a) NCEP1(black line)/NCEP2(blue line) vs. RICH (red line), (c) MSU (red line), and (e) GOZ (red line). (b) ERA-interim data (black line) vs. RICH (red line), (d) MSU (red line), and (f) GOZ (red line). The straight lines are the corresponding linear trend of tropical-averaged CPTT for each dataset from 1981 to 2010. The corresponding correlation coefficients are shown in the top-right corner of each panel.

In this analysis, the meteorological fields (temperature, height, and winds) are based on monthly mean National Center for Atmospheric Research first generation (NCEP1) reanalysis data for the period 1951–2010 (http://www.esrl.noaa.gov/psd/data/reanalysis/reanalysis.shtml). Figure 9 shows the variations in the tropical-averaged CPTTs since the 1980s based on National Center for Atmospheric Research first (NCEP1) and second (NCEP2) generation data, compared with those from the Radiosonde Innovation Composite Homogenization (RICH)^33^ (Fig. 9a), the Microwave Sounding Unit (MSU) and the Advanced Microwave Sounding Unit (AMSU) (https://climatedataguide.ucar.edu/climate-data/msuamsu-atmospheric-temperature-climate-data-record-remote-sensing-systems-rss.) (Fig. 9b), and the Global Ozone Chemistry and Related trace gas Data Records for the Stratosphere (GOZ) (http://disc.gsfc.nasa.gov/datacollection/GozSmlpT_V1.shtml) (Fig. 9c). It should be noted that the CPTT from MSU data is the brightness temperature of the lower stratosphere, which basically represents the variations in CPTT. The CPTT variations from NCEP1 data are in good agreement with NCEP2 and observed CPTTs variations. In our study, we analyzed the spatial distribution of the tropical CPTT from 1951 to 2010. Since there are no NCEP2 data from 1951 to 1978 and the observed data do not include complete information on spatial distributions of the CPTT, we used NCEP1 data. The variations in CPTT from ECMWF reanalysis since the 1980s is not in good agreement with observations (Fig. 9b, d and f). First, the correlations of tropical-averaged CPTTs between observations and NCEP reanalysis are much stronger than that between observations and ECMWF reanalysis. Second, the trends of tropical-averaged CPTTs from observations and NCEP reanalysis are positive, while the trend from ECMWF reanalysis is negative. This means that tropopause temperature in ECMWF reanalysis may have biases, but the NCEP1 tropopause temperature is suitable for this study.

### Model and simulations

The CAM3 model has a longitude–latitude resolution of 2.8° × 2.8° with 26 levels extending from the surface to 4 hPa and a vertical resolution of about 2 km in the tropopause region. Previous studies have shown that the CAM3 can simulate a relatively realistic tropopause and that the common tropopause cold bias problem has been almost eliminated in the model^34^. The SST used in experiments is observed data^35^. All experiments were run for 33 years, where the first 3 years are spin-up and only the remaining 30 years are used for the analysis.

### Statistical significance of correlations

Following Li et al.^36^,^37^, the statistical significance of the correlation between two auto-correlated time series was determined via a two-tailed Student's *t*-test using the effective number (*N^eff^*) of degrees of freedom (DOF)^38^, which can be estimated by: 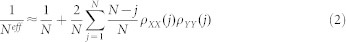
where *N* is the sample size, and *ρ_XX_* and *ρ_YY_* are the autocorrelations of two sampled time series, *X* and *Y*, at the time lag *j*, respectively.

### Separating the CPTT interannual variations

Following Feng et al.^39^, the interannual variations in the tropical-averaged CPTT was split into two components as follows: 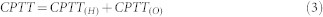

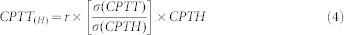

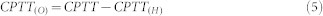
Here, CPTT_(H)_ is caused by the interannual variations in CPTH (represented by a linear fit of the CPTH interannual variations to CPTT interannual variations); CPTT_(O)_ is the part of CPTT interannual variations that is unrelated to CPTH interannual variations; *r* denotes the correlation coefficient between the interannual variations in CPTT and CPTH; and σ(CPTT) and σ(CPTH) are the standard deviations of CPTT interannual variations and CPTH interannual variations, respectively. This method is identical to the semi-partial correlation method, and separates the CPTT interannual variations into two parts: one related to the CPTH interannual variations, and one to other processes.

### Calculation of the overshooting number and its composite anomalies

The overshooting number refers to the monthly count that the overshooting of convection can reach 15 km, based on TRMM data. For further details regarding the calculation, see Liu and Zipser^40^.

Months with El Niño Modoki events are defined as those with Modoki index values equal to or greater than +0.5°C. Months with canonical El Niño events are defined as those with NINO3 index values equal to or greater than +0.5°C. The time range of overshooting number data is from 1998 to 2010. Three strong and continuous El Niño Modoki events (06/2002–04/2003, 06/2004–04/2005, and 09/2009–04/2010) and two strong and continuous canonical El Niño events (01/1998–06/1998 and 08/2006–02/2007) were selected for this study. All events were used to make composite maps of the overshooting number composite anomalies.

### NINO3 and Modoki indices

The monthly NINO3 index and the Modoki index were used to identify monthly occurrences of canonical El Niño events and El Niño Modoki events, respectively. The NINO3 index is defined as the area mean SSTA over the region (5°S–5°N, 150°–90°W), while the Modoki index is defined as follows^28^: 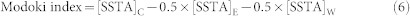
where the subscripted brackets represent the area mean SSTA over the central Pacific region ([SSTA]_C_: 10°S–10°N, 165°E–140°W), the eastern Pacific region ([SSTA]_E_: 15°S–5°N, 110°–70°W), and the western Pacific region ([SSTA]_W_: 10°S–20°N, 125°–145°E). SSTs are based on Met Office Hadley Centre SST (HadISST) data.

## Author Contributions

J.L. and F.X. designed the study and contributed to data analysis, interpretation and paper writing. W.T., Y.L. and J.F. contributed to the discussion and interpretation of the manuscript. All authors reviewed the manuscript.

## Figures and Tables

**Table 1:** Annual tropical (15°N–15°S) averaged CPTH, CPTT, and ω_100_ of simulations (E1 and E2). The value in parentheses is the difference between E2 and E1 (i.e., E2–E1)

Exp	CPTH (km)	CPTT (°C)	ω_100_ (10^−4^ Pa/s)
E1	16.6	−78.5	−4.984
E2	16.7 (0.1*)	−79.4 (−0.9*)	−4.991(−0.007)

*significant at the 95% confidence level.
